# Advancing Precision Nutrition Through Multimodal Data and Artificial Intelligence

**DOI:** 10.1002/advs.202521111

**Published:** 2026-02-11

**Authors:** Yuanqing Fu, Ke Zhang, Zelei Miao, Gaoyi Yang, Yujing Huang, Ju‐Sheng Zheng

**Affiliations:** ^1^ Affiliated Hangzhou First People's Hospital School of Medicine Westlake University Hangzhou China; ^2^ Zhejiang Key Laboratory of Multi‐Omics in Infection and Immunity School of Medicine Westlake University Hangzhou China; ^3^ School of Future Biomedicine School of Life Sciences Westlake University Hangzhou China

**Keywords:** artificial intellengence, brain connectome, genome, gut microbiome, precision nutrition

## Abstract

Interindividual variability in metabolic responses to diets complicates the relationship between nutrition and metabolic health, which highlights the existence of metabolic heterogeneity across populations. This variability challenges the conventional “one‐size‐fits‐all” approach to dietary recommendations and underscores the need for precision nutrition. In the current era, characterized by breakthroughs in sophisticated data collection technologies, the explosion of big data, and progress in artificial intelligence, the implementation of precision nutrition is becoming increasingly feasible. This review aims to summarize potential sources of metabolic heterogeneity from the angle of the host genome, gut microbiome, and brain connectome to explore the implications of their interactions with diet. Furthermore, we discuss the application of artificial intelligence in leveraging multimodal data for predicting individualized dietary responses. Aggregating data on host genetics, gut microbes, and brain activity profiling offers profound insights into the personalized response to diets. We also highlight the development of individual‐specific predictive models that combine n‐of‐1 study designs with advanced wearable technologies and machine learning algorithms, thereby placing the individual at the center of nutritional decision‐making. Finally, this review summarizes current challenges in the field and outlines potential directions for advancing precision nutrition.

## Graphical abstract

Individual responses to food vary dramatically, challenging traditional dietary advice. This review explores how the unique genetic makeup, gut microbiome, and brain activity shape host metabolic health. We examine how artificial intelligence integrates these multimodal data to predict individualized dietary needs, moving beyond one‐size‐fits‐all recommendations toward personalized nutrition strategies.

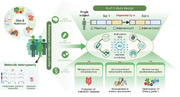

## 1 Introduction

It is widely recognized that poor diet quality contributes substantially to metabolic diseases, including obesity, type 2 diabetes, and cardiovascular diseases [1]. However, interindividual variability in metabolic responses to diets persists, complicating the mechanistic relationships between diet and metabolic health. This metabolic heterogeneity therefore challenges traditional “one‐size‐fits‐all” nutritional guidelines and calls for precision nutrition [2, 3].

Achieving precision nutrition requires accurate, quantifiable, mechanistic, and molecular insights into the sources of metabolic heterogeneity [4]. Omics technologies are widely employed to generate high‐dimensional data that capture informative variations in genetics, epigenetics, proteomics, metabolomics, brain connectome, and gut microbiome. These data enable more accurate estimates of individual dietary requirements than ever, along with improved dietary recommendations and interventions [5, 6, 7, 8, 9, 10, 11]. For example, nutrigenetics refers to the investigation of gene‐diet interaction, aiming to provide personalized dietary advice given individual genetic background [5, 6]. Similarly, the gut microbiome structure and composition are unique to each individual and may determine how we respond to dietary exposures [7, 8, 9]. Functional brain connectome is another robust and reliable “fingerprint” that accurately predicts individual cognition and behavior [10, 11]. Together, these examples highlight that the personalized genetic makeup, gut microbiome, and high variability in brain connectome potentially account for varied responses to specific foods and nutrients. Consequently, the optimal diet for one person may differ significantly from that of another. Nowadays, private companies have started to offer genetic and microbiome testing to the public to customize diets, which opens the door to creating individualized eating plans based on a person's genome and gut microbiome.

Deciphering how genetics and gut microbiota shape individual metabolic responses to diet is methodologically challenging. Artificial intelligence (AI) technologies, increasingly applied to manage and analyze these high‐dimensional omics data, offer promising solutions. Advanced computational approaches facilitate dimensionality reduction, standardization, and normalization of multi‐omics data, while also enabling exploration of relationships both within individual omics levels and across distinct omics layers [12, 13]. While AI offers insights into the solutions to address methodological challenges for precision nutrition, the limited extrapolability of the model requires further investigation [3].

In addition to the accurate estimation of personalized biological responses, higher‐resolution data support the quantification of dietary exposures, which is another essential factor allowing a mechanistic interpretation of nutritional effects. Food frequency questionnaires (FFQ), food diaries, and 24h‐dietary recalls are the three main traditional methods of dietary assessment. However, their limitations are well‐recognized, including susceptibility to recall bias, subjective estimation errors, and restricted reproducibility [14]. Consequently, it is critical to develop objective, reproducible methods to quantify individual dietary habits. The gut, as the primary site of digestion and absorption, offers a unique window into dietary assessment. Gut microbiota are directly exposed to ingested food and nutrients, making fecal metagenome, metabolome, and metaproteomic profiles as powerful tools for assessing dietary intakes and investigating diet‐microbiome interactions [15]. Critically, the integration of AI empowers researchers to analyze these complex, high‐dimensional multi‐omic datasets. The AI‐driven computational framework is essential for developing robust, objective algorithms capable of predicting host dietary consumption and nutritional status directly from microbial signatures [15, 16].

With rapid technological advancements, researchers can now explore an individual's unique genetic background, gut microbiome, brain imaging biomarkers, and environmental exposure profile, making comprehensive and data‐intensive biomedical characterization of individuals feasible. These advances pave the way for addressing nutritional questions at the individual level. Apart from the application of AI in the analysis of multi‐omics data, a lack of innovative study designs poses another significant challenge to conducting precision nutrition research at the individual level. Recently, a single‐subject paradigm, termed “n‐of‐1 trials”, has been increasingly applied to determine whether a specific individual is responding to a particular intervention. This study design compares the response to different interventions within individuals and assesses the variability in these responses by inferring the true effect from random errors, based on repeated cycles of interventions and measurements. The innovative paradigm shows promising potential for integration with emerging biomedical and AI technologies.

Here, we first present an overview of the sources of metabolic heterogeneity and the complex interactions between these factors and diet, which lay the foundation for the concept of precision nutrition. Additionally, we focus on the current status of AI applications in precision nutrition research throughout this review and discuss existing challenges and methodologies (e.g., n‐of‐1 trials) in conducting precision nutrition trials. Finally, we propose the concept of individual‐specific modeling that integrates n‐of‐1 trial designs with AI to further advance the field.

## 2 Implications of Metabolic Heterogeneity in Personalized Dietary Responses

Personalized dietary responses are increasingly recognized as a critical aspect of human metabolism, characterized by interindividual variability in reactions to identical dietary interventions. A landmark study demonstrated that individuals consuming the same portion of white bread exhibited blood glucose spikes varying by over 400% [17]. This heterogeneity is particularly significant given the global challenge of managing postprandial glycemic responses (PPGRs) [17, 18, 19].

**FIGURE 1:**
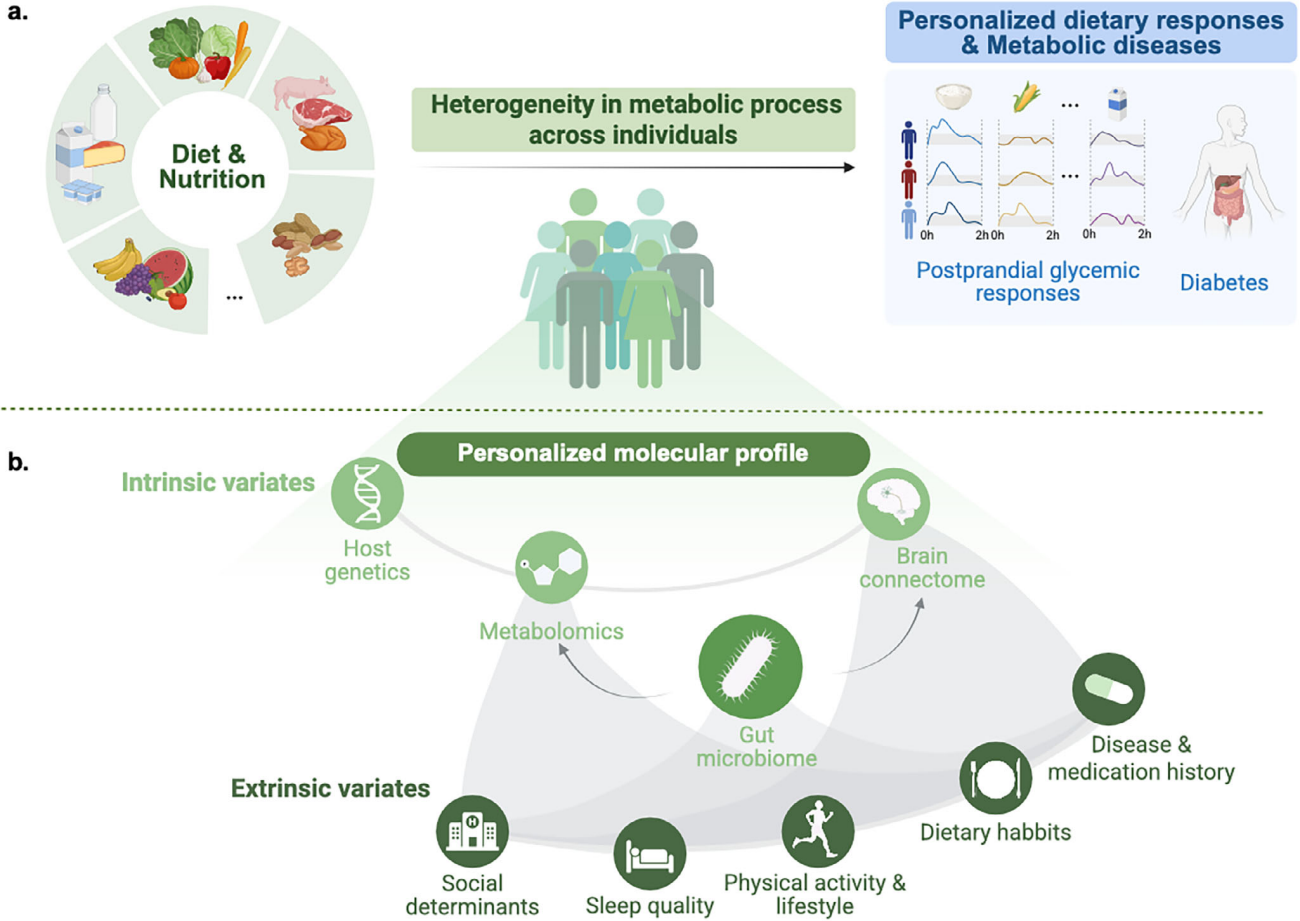
Heterogeneity in metabolic response to diet and associated biological determinants. (a) The upper panel depicts that relationships between diet and metabolic health are complicated by heterogeneity in the metabolic process. (b) The individual‐specific intrinsic and extrinsic factors that contribute to variability in dietary responses and heterogeneity in metabolic health.

Elevated PPGRs represent a widespread epidemic and serve as a major risk factor for prediabetes and type 2 diabetes [18] (Figure 1a). Moreover, the heterogeneity in PPGR extends to other standardized carbohydrate‐rich meals, where even with identical carbohydrate intake, the magnitude of PPGRs differs markedly across individuals [20]. For example, while rice typically induces the highest glycemic response, significant interpersonal variations in PPGRs persist. Moreover, the ranking of PPGR to test meals varies at the population level, with each individual exhibiting their highest response to a specific meal type [20]. Such metabolic heterogeneity challenges current one‐size‐fits‐all dietary guidelines, which are typically formulated based on population‐level averages that mask interindividual heterogeniety.

Genetic variations are recognized as a potential contributor to metabolic heterogeneity observed in dietary responses (Figure 1b). A recent study highlighted that Asian participants were more likely to be rice‐spikers [20], suggesting that both genetic background and dietary habits could shape specific dietary metabolic responses. Among the genetic factors, variations in the genes for apolipoprotein (Apo) A‐I, Apo A‐IV, Apo B, and Apo E have been shown to influence lipid metabolism in response to dietary changes [21]. In addition, specific genetic variations affect how the body processes nutrients, which directly impact postprandial dietary responses [22]. A notable example is the *AMY1* gene, which encodes salivary amylase, a key enzyme that breaks down starch in carbohydrates. The copy number of the *AMY1* gene exhibits a positive correlation with enzyme expression levels, modulating the body's ability to metabolize carbohydrates [23]. Beyond single genetic mutation, polygenic scores integrating hundreds of genetic variants have been shown to predict individualized low‐density lipoprotein (LDL) cholesterol responses to dietary fat reduction, underscoring the complex genetic architecture underlying metabolic heterogeneity [24]. Despite these advances, the promise of nutrigenetics, where genetic information is used to predict responses to dietary inputs, remains limited by the relatively small sample sizes of many studies [25]. Continued efforts to expand sample sizes and broaden the scope of dietary interventions will be critical for advancing the field [26].

Environmental factors, encompassing dietary patterns, physical activity, and early‐life exposures, further explain metabolic heterogeneity in dietary responses [27]. Among these, the gut microbiome emerges as a central integrator of environmental influences, acting as a “metabolic interface” between diet and host physiology [28] (Figure 1b). A critical function of the gut microbiome lies in its regulation of host metabolic processing for diverse dietary components (e.g., fibres, polyphenols, bile acids), which fundamentally shapes metabolic outcomes and underpins interindividual heterogeneity. For instance, a cohort study of 307 men demonstrated that adherence to a Mediterranean diet was associated with a lower risk of cardiovascular disease [29]. This effect was primarily observed in individuals lacking the gut bacterium *Prevotella copri*. Conversely, carriers of *P. copri* did not exhibit this benefit despite similar dietary adherence. Moreover, a controlled intervention study revealed that participants who improved their glucose metabolism after consuming dietary fibre had significantly higher levels of *P. copri* [30]. These individuals also showed a greater capacity to ferment complex polysaccharides than non‐responders. The above evidence directly links the bacterium's metabolic activity to divergent responses to diet. Importantly, such microbial factors are integral to predictive models of PPGRs. For example, by using the stochastic gradient boosting regression, researchers integrated blood parameters, diet, anthropometrics, physical activity, and microbiome data to predict PPGRs [17]. In a blind randomized controlled trial, researchers used this algorithm to design personalized dietary interventions for each participant. Results showed that these tailored diets significantly reduced PPGRs while inducing consistent shifts in gut microbiota compositions. These findings collectively highlight how the gut microbiome, through its metabolic activity, contributes to hosts’ metabolic heterogeneity and drives individualized responses to dietary components.

## 3 The Host Genetics, Gut Microbiome, and Brain Connectome in Personalized Dietary Response for Precision Nutrition

The emerging field of precision nutrition integrates multimodal data to predict and optimize dietary responses. Among intrinsic factors, we focused on gene–diet interactions, which have been extensively studied in the field of nutrition over recent decades [6, 31]. With regard to environmental factors, the gut microbiome has emerged as a key area of investigation, serving as a primary mediator between dietary intake and host metabolism [32]. As such, it plays a central role in explaining the heterogeneous responses to diet observed across individuals. Furthermore, advances in data collection and processing technologies have led to growing interest in multimodal data, including 2D or 3D brain image data, which hold substantial promise for explaining the heterogeneity of dietary choice and metabolic responses [33]. In this context, we also prioritize the implications of brain connectome architecture and its integration with artificial intelligence algorithms for advancing precision nutrition in this review. Taken together, by leveraging these interconnected biological layers, precision nutrition aims to develop tailored dietary strategies that maximize health outcomes.

### 3.1 Host Genetics: Gene‐Diet Interactions and Health Outcomes

**FIGURE 2:**
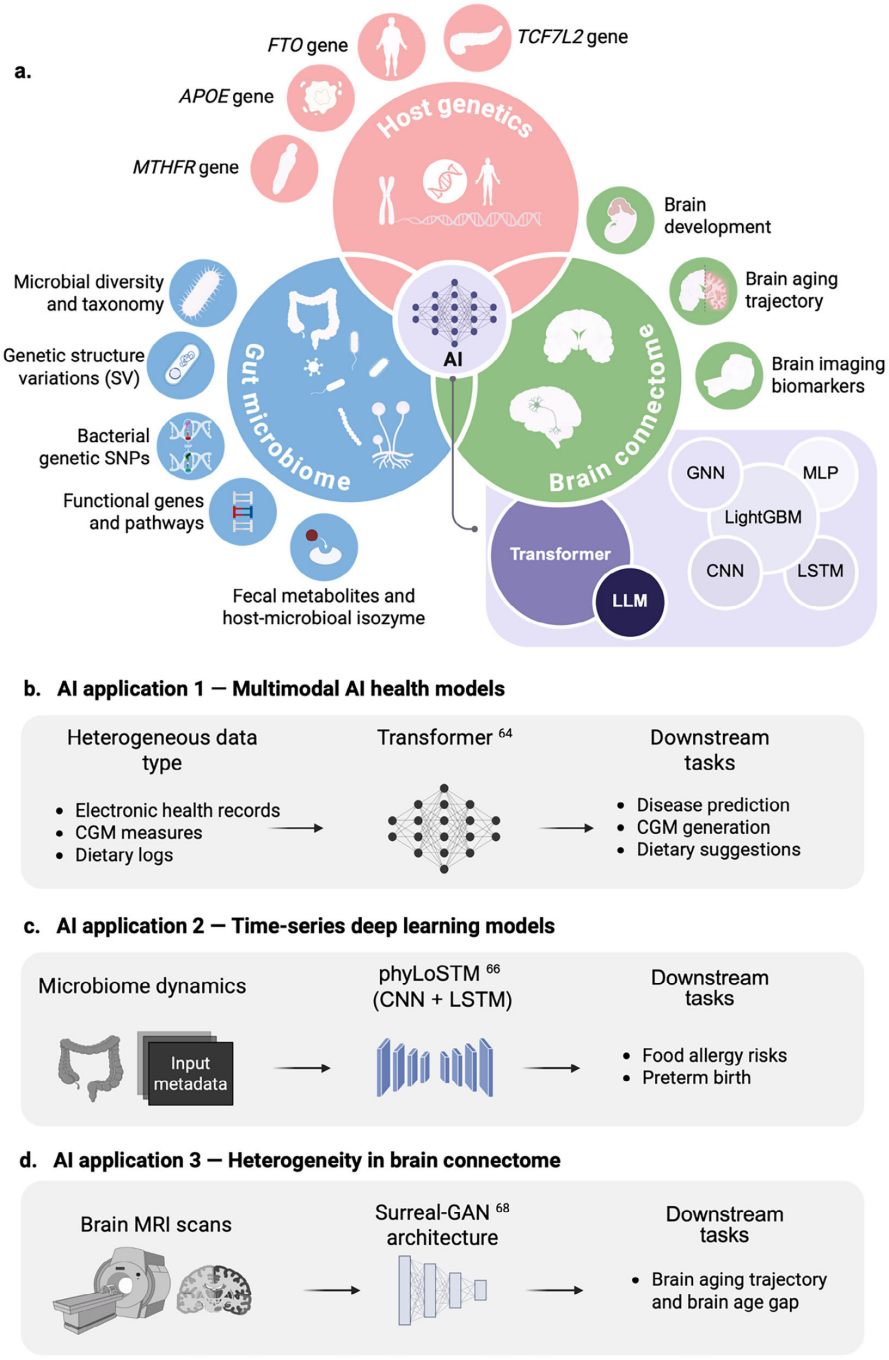
Systemic illustration of artificial intelligence and multimodal database. (a) Integration of multi‐omics and AI for modeling. The methods that have not been widely used in prior studies on precision nutrition are highlighted with a darker shade, whereas those commonly employed in previous literature are grouped together and indicated with a lighter shade. (b–d) Three related cases represent the applications of artificial intelligence in multimodal health modeling. Abbreviations: SVs, structure variations; MRI, magnetic resonance imaging; SNPs, single nucleotide polymorphisms; CNN, convolutional neural network; LSTM, long short‐term memory; CGM, continuous glucose monitoring; AI, artificial intelligence.

Host genetic variations can shape individual nutritional requirements and physiological responses to diet, driving stratified health outcomes with profound implications for clinical practice (Figure 2a). This gene‐diet interplay manifests through distinct biological mechanisms. First, genes influence the metabolism and utilization efficiency of specific nutrients. A classic example is the polymorphism in the methylenetetrahydrofolate reductase (*MTHFR*) gene, which regulates folate one‐carbon metabolism and planar cell polarity, which can subsequently elevate risks of neural tube defects and cardiovascular diseases [34, 35]. Individuals with these variants would have different folate requirements to maintain health. Another key example involves the apolipoprotein E (*APOE*) gene, for which different alleles are strongly associated with blood lipid levels and cardiovascular disease risk. Research has shown that a Mediterranean diet can effectively modulate postprandial hypertriglyceridemia in patients with coronary heart disease through its interaction with the *APOE* gene [36]. Furthermore, genome‐wide scans illustrate how natural selection adapts populations to traditional diets, exemplified by the *FADS* gene variants in Inuit populations that enhance metabolism of omega‐3 fatty acids abundant in marine diets [37].

Interestingly, modern dietary exposures and lifestyle factors can either amplify or mitigate an individual's genetic predisposition to disease. A well‐documented example is the interaction between sugar‐sweetened beverages and obesity risk [38]. Among individuals with a high genetic susceptibility to obesity, consumption of sugar‐sweetened beverages exacerbates this risk in a dose‐dependent manner. More importantly, modifiable lifestyle factors can powerfully counteract genetic risk. A large‐scale study demonstrates that even among individuals at high genetic risk for coronary artery disease, adherence to a healthy lifestyle (non‐smoking, no obesity, regular physical activity, healthy diet) was associated with a nearly 50% reduction in coronary artery disease incidence compared to those with an unfavorable lifestyle [39]. Collectively, these paradigms highlight the critical role of gene‐environment interactions, particularly with diet and lifestyle, in determining an individual's health trajectories. This underscores the necessity for precision nutrition strategies that tailor dietary and lifestyle recommendations to an individual's genetic background to achieve optimal health outcomes.

### 3.2 Gut Microbiome: Gut Microbial Stability and Plasticity in Personalized Dietary Response

The interplay between microbial dynamics and adaptive plasticity is one of the factors that underpins personalized responses to dietary interventions, drug administrations, and other environmental exposures (Figure 2a). Under standardized meal test conditions, the gut microbiome explains significantly more interindividual variations in postprandial triglyceride and insulin responses than dietary composition itself, highlighting its potential as a key determinant for predicting personalized metabolic outcomes [2]. On the other hand, although the gut microbial compositions fluctuate with the environment at the abundance level, their genetic structure variations (SVs) exhibit long‐term stability and enable the identification of individual specificity, establishing a foundational “microbial fingerprint” [40]. Moreover, gut microbial SVs have crosstalk with the host genome, revealing host‐microbiome co‐adaptation and co‐divergence [41]. For instance, host *ABO/FUT2* genotypes drive secretion of mucosal GalNAc‐terminated antigens, which selectively enrich gut microbiome strains carrying GalNAc‐utilization genes (e.g., *Faecalibacterium praus*.). These functionally adapted microbes, in turn, influence host cardiometabolic health [41]. A metagenome‐wide association study from 7,190 healthy participants discovered 1,358 host BMI‐associated bacterial single‐nucleotide polymorphisms (SNPs), underlying the importance of understanding interpersonal differences at microbial nucleotide level, as well as their potential health implications [42]. Transitioning from “abundance” to “microbial genetics” builds a bridge that links the structural taxonomy to its underpinning biological functions.

The gut microbiome serves as a dynamic regulator of host metabolism, with its interactions with diet exerting significant and heterogeneous effects on individual physiological responses. A growing body of research establishes a multidimensional evidence chain demonstrating the complexity of this regulatory network. For example, an observational study of 1,368 deeply phenotyped individuals revealed that interactions between the gut microbiome and diet significantly influence interindividual variations in the plasma metabolome [43]. This finding bridges environmental exposures with molecular regulatory pathways underlying host metabolic phenotypes. Meanwhile, parallel diet‐microbiome‐disease pathways exist for trimethylamine N‐oxide (TMAO), a metabolite derived from microbial processing of red meat‐associated choline. Elevated TMAO correlates with atherosclerosis and thrombosis risks. Recent research further identifies the *gbu* gene cluster as a key microbial determinant enriched in high‐TMAO producers, accelerating the conversion rate of L‐carnitine to TMAO [44]. Despite modifiability via antibiotics or enzyme inhibitors, clinical translation remains limited, with current practice favoring generic red meat restrictions over microbiome‐informed strategies [45]. Notably, identification of individuals with elevated TMAO production capacity, achievable through cost‐effective methods like the oral carnitine challenge test, could enable precision nutrition interventions [46]. This underscores the critical gap in harnessing microbial biomarkers to optimize dietary recommendations for at‐risk populations.

The gut microbiome further stratifies treatment responses to pharmaceuticals. For instance, Sitagliptin, a conventional inhibitor of the host dipeptidyl peptidase 4 (DPP4) for the treatment of type 2 diabetes, exhibits distinct efficacy across participants [47]. By integrating activity‐based enzyme screening and cultivation‐based characterization, Wang et al. found the microbial‐host isozyme that can not be inhibited by this drug effectively [47]. Under a high‐fat diet‐induced gut hyperpermeability in the mice model, microbial DPP4 translocates systemically, reducing active glucagon‐like peptide‐1 and disrupting glycemic homeostasis. In short, the gut microbiome serves as a dynamic modulator for personalized health outcomes by interacting with dietary exposures and pharmaceuticals, explaining interindividual heterogeneity in metabolic benefits and treatment efficacy through host‐microbe molecular crosstalk.

### 3.3 Brain Connectome: Implications of the Individual Variations in Brain Connectome Architecture for Precision Nutrition

The brain imaging biomarkers, typically characterized by the brain connectome, a dynamic network of neural connections, exhibit profound individual variability in architecture and functional dynamics [11, 48]. This heterogeneity fundamentally shapes responses to nutritional stimuli across the lifespan, from neurodevelopment to aging [49]. Advanced neuroimaging techniques (e.g., functional magnetic resonance imaging (fMRI) with high spatial resolution, electroencephalography (EEG) with high temporal resolution) facilitate quantitative analyses of individual‐specific neural activities. Such tools are pivotal for understanding how the personalized brain mediates nutritional impacts on cognitive and physiological systems.

In psychiatric disorders such as depression and anxiety, hierarchical clustering for brain imaging data has identified biologically distinct subgroups through individualized circuit scores [48]. These scores reveal that disease manifestation is intrinsically tied to personal neurobiology, challenging one‐size‐fits‐all diagnostic paradigms. Similarly, during neurodevelopment, functional network topography varies substantially among children. For instance, fronto‐parietal network connectivity in early life predicts cognitive abilities, underscoring the functional consequences of connectomic individuality [50]. These findings advocate for a paradigm shift toward precision neuroscience, where interventions are tailored to an individual's neural architecture.

Individual variations in brain connectome architecture across populations present a novel framework for understanding dietary decision‐making processes within the scope of precision nutrition research. Large‐scale neuroimaging initiatives, such as a reference database tracking functional connectivity in 42,428 individuals, provide normative baselines to quantify individual deviations [51]. Brain functional connectivity undergoes nonlinear changes across the lifespan, with aging trajectories shaped by differential exposure to lifestyle factors, genetics, and environmental influences [49]. To take appetite and eating behaviours for instance, eating behavior can be regulated through hypothalamic “hunger neurons” and the orbitofrontal cortex (OFC), which acts as a multisensory integrator to process visual/gustatory signals in modulating reward circuitry [52]. In diabetic populations, high‐calorie food cues enhance OFC activation [53, 54], while obesity reduces GABAergic inhibition in rodents, causing OFC hyperactivity [55]. Dysregulated brain connectivity may underlie binge eating disorders [56]. Moreover, interindividual variation in the connectivity strength between ventromedial and dorsolateral prefrontal cortex correlates with optimal decision making about food consumption [57]. By mapping neural substrates of eating behaviors, such integrative approaches enable personalized interventions targeting gut‐brain communication pathways.

The role of brain activities as a mediator of nutritional effects on neurocognitive health is increasingly evident. Neural signatures not only predict cognitive trajectories and disease risk but also likely modulate nutrient metabolism, dietary intervention efficacy, and neurobiological resilience. For example, interindividual differences in reward circuitry connectivity may explain variable responses to sugar or fat intake, while default mode network dynamics could influence stress‐related eating behaviors [58]. To transcend population averages, recent work has generated a precision functional atlas of personalized brain topography using data from >9,900 individuals across ages and cohorts [59]. This atlas enhances reproducibility in neuroimaging and guides neural interventions such as transcranial magnetic stimulation, where targeting individual‐specific networks improves therapeutic outcomes. Such tools exemplify how neural profiling can refine nutritional strategies: since neural network topology predicts responses to brain stimulation, it may also forecast metabolic responses to dietary interventions.

Taken together, advanced neuroimaging and machine learning techniques now enable the mapping of individual‐specific neural architectures, revealing how brain network topology predicts responses to dietary interventions and disease risk. This precision neuroscience framework holds particular promise for heterogeneous conditions like obesity and metabolic syndrome, where one‐size‐fits‐all dietary guidelines often fail. By integrating brain connectome profiling into nutritional strategies, we may develop tailored interventions that optimize both neuroprotection and metabolic health based on an individual's unique neural architecture.

## 4 Artificial Intelligence in Applications of Multimodal Data

Artificial intelligence is revolutionizing the analysis of multimodal data in health and nutrition. By integrating diverse data sources from genomics and microbiome sequencing to electronic health records, medical imaging, and wearable biosensors, AI systems are uncovering complex relationships between host genetics, gut microbiota, lifestyle factors, and disease states. Advanced frameworks, including large language models, deep neural networks, and hybrid architectures like phyLoSTM, enable dynamic health profiling, personalized aging assessments, and real‐time metabolic simulations. In precision nutrition, AI‐powered tools enhance dietary assessment accuracy, correct self‐report biases, and optimize dietary patterns through objective biomarkers and data‐driven feature prioritization.

### 4.1 AI‐Powered Applications of Multimodal Data in Health Profiling

The expanding availability of biomedical data from diverse sources (e.g., large biobanks, electronic health records, medical imaging, wearable devices, and ambient biosensors), coupled with declining costs for genome and microbiome sequencing, has accelerated the development of multimodal AI systems [60]. These systems are uniquely positioned to unravel the intricate interplay of factors shaping human health and diseases.

AI is increasingly leveraged to identify relationships between host genetics, the microbiome, and various health conditions (e.g., diabetes, cardiovascular disease, cancer). By integrating machine learning with electronic health records and/or genomic data, this framework dynamically assesses nutritional deficiencies, disease risks, and stratifies patients into actionable risk categories [61]. This employs a spectrum of techniques to improve personalized health management, from conventional machine learning to deep neural networks [62] (Figure 2a and Table 1). Recently, a large language model (LLM)‐based aging assessment framework was developed, which integrates health examination reports with lifestyle information to estimate overall and organ‐specific aging (Table 1) [63]. Notably, the LLM‐predicted overall age demonstrates a significant association with all‐cause mortality, outperforming other aging proxies, including telomere length and the frailty index. Furthermore, a multimodal framework termed GluFormer, utilizing self‐supervised learning (Table 1), trained on dietary intake and continuous glucose monitoring data, demonstrates superior performance in predicting disease onset [64] (Figure 2b). Specifically, participants log their food and drink consumption, including information such as types of food, serving sizes, and the times of ingestion, on a proprietary smartphone app. Leveraging with CGM data, the GluFormer can stratify prediabetic patients into progression or stability. This model serves as a personalized digital twin, which dynamically integrates multi‐omics data, lifestyle factors, and real‐time health metrics to simulate individual metabolic responses. It enables the tailoring of precision nutrition interventions and provides a scalable platform for personalized health management.

**TABLE 1:** A comparative summary of core artificial intelligence terminology.

Term	Typical input	Typical output	Underlying logic
Machine learning, ML [73]	Structured or unstructured data (e.g., numbers, text, images).	Predictions, classifications, or decisions based on the input data.	Algorithms that learn patterns from data to make predictions without being explicitly programmed for the task.
Deep neural networks, DNN [62, 67]	Large datasets of various types, such as images, text, or sound.	Learned features, classifications, or generated data.	A class of machine learning models with multiple layers of interconnected nodes (“neurons”) that learn hierarchical representations of data.
Deep‐representation learning [68]	Raw, often unstructured, data.	Meaningful, hierarchical features or vector representations of the input data.	Automatically discovers and learns a hierarchy of features from data, transforming it into a more abstract and useful representation for tasks like classification.
Convolutional neural network, CNN [69, 84]	Grid‐like data, most commonly images.	Class labels, object detections, or feature maps.	Applies filters across the input data to learn spatial hierarchies of features, making it highly effective for image analysis.
Recurrent neural networks, RNN [66]	Sequential data, such as text or time series.	A sequence of outputs or a single prediction.	Utilizes feedback loops to maintain an internal state or “memory,” allowing it to process sequences of data and capture temporal dependencies.
Long short‐term memory, LSTM [66]	Sequential data, particularly long sequences.	A sequence of outputs or a prediction based on the sequence.	An advanced type of RNN with a gating mechanism to control the flow of information, enabling it to learn long‐term dependencies in data more effectively than simple RNNs.
Self‐supervised learning [64]	Large amounts of unlabeled data.	A pre‐trained model with learned representations of the data.	A model learns from the data itself by creating its own labels from the input, for instance, by predicting a missing part of the data.
Large language model, LLM [63]	A prompt, which can be a question, instruction, or a piece of text.	Human‐like text, code, or other forms of sequential data.	A very large deep learning model, often based on a transformer architecture and trained on vast amounts of text using self‐supervised learning, to understand and generate language.

Assessing temporal dynamics of omic profiling is another critical perspective to understand biological processing, particularly in microbial sequencing studies. Traditionally, longitudinal approaches often employ feature‐by‐feature univariate frameworks that assume independence between individual features [65]. These community‐level adaptations manifest as coordinated longitudinal dynamic shifts that cannot be fully captured through isolated feature analyses. Therefore, a comprehensive understanding of microbiome dynamics requires not only tracking temporal changes but also elucidating the physical crosstalk and functional synergies between community members. For example, long short‐term memory networks (LSTM), a specialized variant of recurrent neural networks, have emerged as powerful tools for modeling temporal dynamics in microbiome studies [66]. Building on this foundation, phyLoSTM represents an advanced architecture that combines convolutional neural networks (CNNs) for spatial feature extraction with LSTM units for temporal sequence analysis (Figure 2c) [65]. This hybrid approach demonstrates superior predictive performance in clinical applications such as food allergy risk assessment and preterm birth prediction [65]. Additionally, the McMLP (Metabolite response predictor using coupled multilayer perceptrons) deep learning architecture leverages microbiome composition with dietary records to predict postprandial dietary responses. Trained on synthetic data and validated across six dietary intervention cohorts, McMLP demonstrated superior accuracy in capturing the temporal dynamics of metabolic shifts compared to conventional machine learning models [67]. It hints that microbiome features encode reliable, time‐sensitive predictive information for personalized nutrition. This integration of temporal omic profiling with AI‐driven modeling exemplifies how dynamic, time‐aware analyses advance precision nutrition beyond static snapshots, directly addressing the core challenge of understanding biological processing through temporal dynamics.

To identify the heterogeneity in brain development and aging trajectory, AI is also a powerful tool to handle the high‐dimensional brain imaging dataset. For example, the Surreal‐GAN framework, a deep‐representation learning (Table 1) method, was developed in the dimensional analyses of resting‐state fMRI data from 49,482 participants. It identifies 5 dominant and distinct brain atrophy patterns, representing personalized profiles of accelerated aging (Figure 2d) [68]. Such resources, combined with AI‐driven tools like CNN‐based “brain age” prediction models, link connectomic divergence to neurodegenerative syndromes [69]. Critically, the “brain age” gap, namely the discrepancy between chronological and predicted brain age, has been serving as a biomarker for resilience or vulnerability to metabolic and cognitive decline. Collectively, these examples demonstrate the promising application of an AI‐powered platform for elucidating the complex relationship between multimodal data and health conditions. Moreover, integration of heterogeneous host genenome, gut microbiome with brain activity profiling possesses great potential to enhance AI‐driven platforms, which offers unprecedented opportunities to decode personalized metabolic states and health trajectories.

### 4.2 Integration of Artificial Intelligence and Omics Data for Enhancing Precision in Dietary Assessment

**FIGURE 3:**
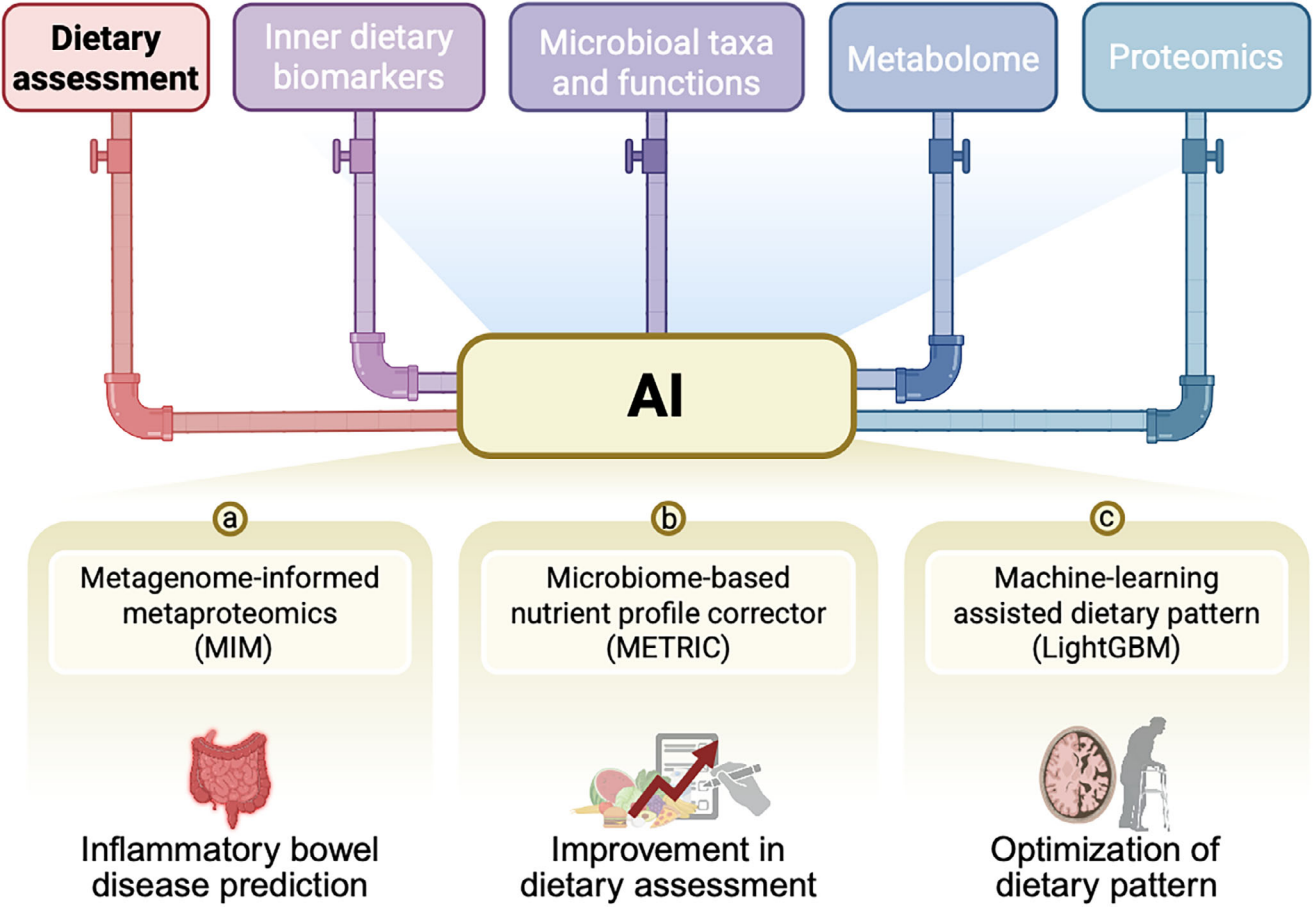
Accuracy promotion of dietary assessment by integrating artificial intelligence and multi‐omics. (a–c) Three cases demonstrating how AI enhances dietary assessment through integration with multi‐omics data. Abbreviation: FFQ, food frequency questionnaire; METRIC, microbiome‐based nutrient profile corrector; MIM, Metagenome‐informed metaproteomics; MORDEN, machine learning‐assisted optimization of dietary intervention against dementia Risk; AI, artificial intelligence; IBD, inflammatory bowel disease.

Precision nutrition requires objective dietary assessment tools to elucidate diet‐health relationships, as current subjective methods (e.g., FFQ) introduce recall bias and limit causal inference [70]. A web‐based self‐administered 24h recall tool (ASA24) represents a cost‐benefit improvement in terms of data collection and management, which contributes to reducing burden in the field [71]. AI‐powered food image recognition shows promise for estimating energy and nutrient intake to reduce dietary assessment errors and increase participants’ adherence, though further refinement of recognition model accuracy is still needed [72] (Figure 3).

Dietary biomarkers from the circulating system or gut are one of the optimal ways to assess internal exposure to diet [14]. Emerging evidence highlights the gut microbiome as a dynamic biomarker for dietary exposure, with multi‐omics approaches offering high‐resolution nutritional phenotypes. Metagenome‐informed metaproteomics (MIM) has emerged as a powerful non‐invasive technique to dissect host‐microbiome‐diet interactions in both murine and human studies [16]. MIM simultaneously maps nutritional exposure landscapes while identifying species‐specific functional dysbiosis, as demonstrated in inflammatory bowel disease studies where suppressed commensal responses to host inflammatory signals were uncovered [16]. With aims of correcting measurement errors brought by recall bias in the self‐reported nutrient assessment, Wang et al. integrate microbiome‐derived signals with the multilayer perceptron model, and provide a novel deep‐learning approach (METRIC, Microbiome‐based nutrient profile corrector) [15]. METRIC is applied to 3 distinct real clinical datasets where noise has been artificially introduced into the nutrient profiles. The results demonstrate that METRIC effectively corrects the nutrient profiles, particularly for nutrients with large errors or those metabolized by gut microbiota. This methodology, integrating AI (e.g., METRIC) with multimodel data, offers a powerful synergy to enhance dietary assessment precision, holding significant promise for advancing precision nutrition research.

AI also enhances dietary pattern optimization through data‐driven feature prioritization. For example, a recent study employed food‐wide association analysis to identify 25 dementia‐associated food groups, then applied LightGBM‐based ranking to distill eight critical components (e.g., leafy greens, berries) into the MODERN (machine learning‐assisted optimization of dietary intervention against dementia risk) score [73]. This AI‐optimized index outperformed the conventional MIND (i.e., Mediterranean‐DASH intervention for neurodegenerative delay diet) diet in dementia risk prediction and revealed novel associations with mental health outcomes [73]. Another AI‐informed diet, a personalized postprandial‐targeting (PPT) diet aimed at lowering postprandial glycemic responses to meals, promoted greater dietary modifications on cardiometabolic outcomes, compared to the conventional Mediterranean diet [19].

These advances collectively demonstrate AI's transformative capacity to address fundamental limitations in dietary assessment methodologies. The convergence of these innovations significantly enhanced measurement precision through objective, multi‐dimensional nutritional phenotyping. Looking forward, clinical translation will require standardized AI frameworks capable of integrating real‐time physiological data from wearable technologies. Equally critical is the validation of these systems across global dietary patterns to ensure cultural and regional applicability. Ultimately, this AI‐driven integration of multi‐omics technologies promises to redefine precision nutrition by establishing microbiome‐informed dietary assessment as a cornerstone of evidence‐based chronic disease prevention strategies.

## 5 Integration of AI with n‐of‐1 Trials Stimulates the Concept of Individual‐Specific Modeling

Despite the popularity and recognition of the concept of “individualized nutrition” or “precision nutrition”, the research methodologies that account for the interindividual variability in treatment response remain underdeveloped. Modern medicine has looked up to the population‐based, parallel‐group, randomized‐controlled trials (RCTs) as its gold standard for guiding clinical decision‐making [74]. But RCTs are designed to assess the average treatment effects for the population at large, without effectively tackling the individual particular characteristics that may modify treatment response or addressing the definitive benefits for any individual participant [75]. In the era of emerging precision nutrition, a single‐subject research methodology that can generate evidence to inform individualized treatment decisions is warranted [14, 76].

**FIGURE 4:**
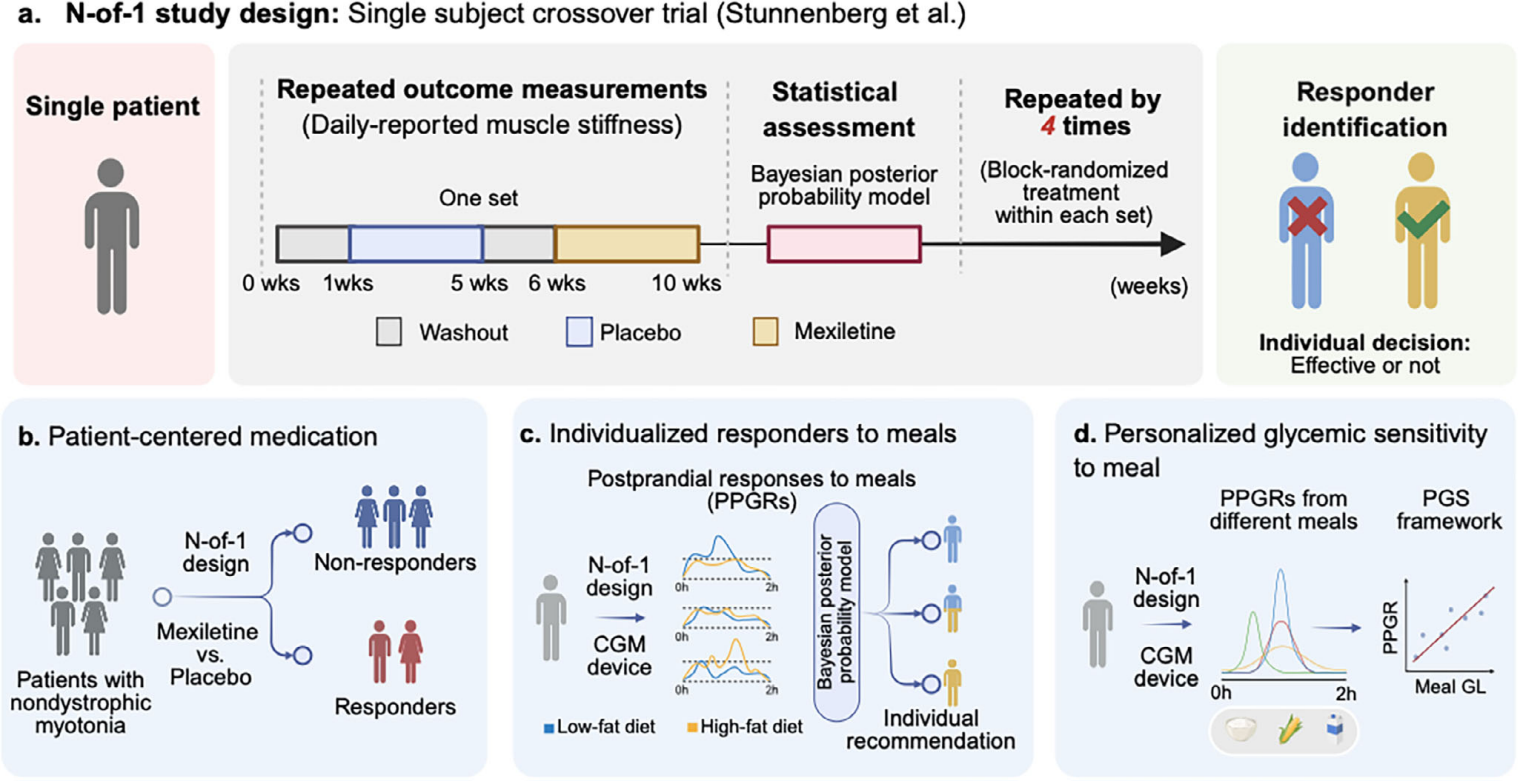
Characteristics of n‐of‐1‐based study design and applications. (a) Graphic illustration of n‐of‐1 study design with wearable device monitoring. (b–d) Three applications of n‐of‐1 study designs in patient‐centered medication and assessment of individualized glycemic responses to meals. Abbreviations: CGM, continuous glucose monitoring; PGS, Personalized glycemic sensitivity index.

Unlike traditional population‐based clinical trials, n‐of‐1 trial focuses on individual responses and thus are a promising methodology and solution that supports single‐subject research. Briefly, n‐of‐1 trials in clinical medicine are multiple crossover trials, usually randomized and often blinded, conducted in a single subject, aiming to compare her/his response to different interventions and assess the variability in these responses (Figure 4a) [77]. For example, Stunnenberg and colleagues conducted a series of placebo‐controlled n‐of‐1 trials of mexiletine in patients with nondystrophic myotonia, which is a rare chronic disease caused by mutations in the skeletal muscle sodium ion (*SCN4A*) or chloride ion (*CLCN1*) channel gene [78]. In this study, each n‐of‐1 trial consisted of 1 to 4 treatment sets, and each set comprised a 4‐week period of mexiletine and a 4‐week period of placebo treatment, with a 1‐week washout in between and 2 weeks for statistical interim analysis at the end. Each patient was randomly assigned to receive mexiletine hydrochloride capsules or placebo capsules in a blind, cross‐over manner within each set, with the treatment randomization performed independently within each patient. The treatment effect was also independently estimated for each patient based on the daily‐reported muscle stiffness within the individual. In this way, they identified patients who responded to the treatment and those who did not, and mexiletine therapy was continued or discontinued accordingly (Figure 4b). This design is particularly appealing for precision nutrition research, as it places the individual at the center of decision‐making, rather than focusing on the “average response” observed in a population. Furthermore, in n‐of‐1 trials, each participant acts as their own control, since statistical analysis relies solely on data collected from that individual under different interventions, independent of group‐level mean values typically derived from a group of participants. Consequently, potential confounding effects arising from interindividual heterogeneity at the group level (e.g., age, sex, BMI, and baseline characteristics) are effectively eliminated. Importantly, n‐of‐1 trials have been classified as providing “level 1” evidence for evaluating treatment efficacy in individual patients [79].

Compared to conventional RCTs, much more data, particularly repeated measurements within each subject, are expected to be collected in n‐of‐1 trials, as valid inference at the individual level is required. The development of electronic health monitoring technologies and wearable devices enables the systematic and detailed collection of data. In our WEMACNUTR [77] and WePrecision study [80], both continuous glucose monitoring (CGM) systems and triaxial accelerometers were applied. Such intensive data collection is essential for researchers to accurately compare the effects of different interventions at an individual level (Figure 4c). For instance, CGM‐derived postprandial metrics could be calculated three times per day over six 6‐day intervention periods, which will yield 108 observed data points throughout three cycles. This strategy of repeated measurements of outcome variables is to address the intraindividual variabilities within a single individual [81]. As suggested in the guideline book of n‐of‐1 trials, usually three crossover intervals were applied in the n‐of‐1 trials [81]. Although there is no conclusive number of how many repetitions of measurements are good enough for n‐of‐1 trials, Stunnenberg and colleagues proposed a simulation‐based strategy to calculate the p‐value and statistical power, evaluating whether or not the designed number of repetitions is enough for controlling the type I and type II errors [78]. Moreover, multi‐omics data (e.g., genome, proteome, metabolome, brain connectiome, and gut microbiome) are emerging as powerful tools for comprehensively profiling individualized characteristics and providing deeper biological insights [82, 83]. However, they also pose significant challenges to data processing and analytical frameworks.

AI‐based solutions have been widely integrated to address the need for processing big data and aiding decision‐making. Taking brain imaging data as an example, hierarchical clustering is one of the unsupervised method which has been applied to identify clinically distinct biotypes in depression and anxiety based on personalized brain circuit scores [48]. AI approaches, such as neural networks and LSTM, are also widely utilized to predict brain age and derived age gap via integrating multimodal brain imaging data, which provide insights for subtypes of brain development, heterogeneity in aging trajectory, as well as stratified risks of dementia [69]. However, most of these machine learning prediction models were still trained on population‐level data and then applied to individuals. This approach is based on the assumption that there are common or shared patterns across populations, which somewhat contrasts with the principles of precision nutrition that emphasize the uniqueness of each individual. Nevertheless, n‐of‐1 trials, which involve intensive data collection within individual participants, offer the potential to integrate single‐subject information directly into model training, thereby aligning more closely with the objectives of precision nutrition. For example, Jansen et al. reported a pioneering work that studied the improvement in performance of lesion quantification methods on magnetic resonance images (MRI) following patient‐specific fine‐tuning, as compared to a pretrained base convolutional neural network (CNN, Table 1). Briefly, they leveraged previously acquired imaging data, in which more than one imaging examination is available for a patient, and employed CNN‐derived methods to extract valuable information from these images, thereby aiming to enhance lesion quantification on neural images of the same patient. In their study, the patient‐specific fine‐tuned CNN outperformed the base CNN, with the median true positive rate increasing from 0.67 to 0.85 for liver metastasis detection [84]. This study suggests the feasibility of incorporating individual‐specific information into model training, rather than relying solely on pre‐existing population data.

Following the individual‐specific modeling strategy, we modeled the personalized relationship between dietary glycemic load (GL) and PPGRs in our WePrecision n‐of‐1 study [80]. Specifically, to address the limitations of population‐averaged models in nutrition research and capture interindividual variations in dietary responses, we implemented an n‐of‐1 framework in the WePrecision studies, establishing a subject‐specific framework fitting the mathematical relationship between GL and PPGR. This led to the concept of the personalized glycemic sensitivity index (PGS), which quantifies metabolic responsiveness to dietary carbohydrates at the individual level. This concept is characterized by individualized modeling using only personal CGM‐meal paired data (Figure 4d) [80]. Moreover, further iterative refinement capability is reserved when new dietary data automatically updates model parameters. This contrasts with static population‐based strategies that ignore intraindividual metabolic evolution. This glycemic sensitivity index is considered a novel and timely concept with potentially important implications for personal and public health [85]. Advanced data treatment and individual‐specific modeling were considered as strengths, making this research compelling. Taken together, the PGS framework provides promising insights that personalized nutrition need not sacrifice scientific rigor. Conversely, it achieves greater precision by embracing biological uniqueness through temporally resolved, subject‐centric modeling.

In summary, the development of the PGS index exemplifies how subject‐specific frameworks can quantify metabolic responses at the individual level, with AI algorithms enabling continuous refinement as new data becomes available. This approach not only maintains scientific rigor but also achieves greater precision by capturing biological uniqueness through temporally resolved, subject‐centric modeling, offering a promising pathway for personalized nutritional interventions.

## 6 Discussion

Precision nutrition is an approach that integrates individual‐level multimodal data to predict how a specific individual will respond to particular foods or diets. In this regard, multimodal data and powerful predictive models are thus two essential components for advancing the field of precision nutrition. Additionally, precision nutrition research also calls for novel designs to help address nutritional questions at the individual level, and the n‐of‐1 design discussed in the present review is likely to become a very promising approach. This methodology focuses on intraindividual variability in response to various interventions and eliminates group‐level confounding factors [77]. It represents a paradigm shift in precision nutrition research, moving beyond population‐averaged approaches to embrace individual‐specific modeling.

Metabolic heterogeneity underlies the varying responses to food across individuals, and harnessing these metabolic heterogeneities is becoming promising with declining costs of multi‐omics data profiling and advancements in real‐time monitoring technologies [2, 86]. Notably, the inherent sparsity of personalized data demands AI‐driven solutions that balance robustness and adaptability. Meta‐learning enables rapid adaptation to individual dietary responses [87], and transfer learning leverages cross‐cohort insights to pre‐train models [88]. Data augmentation and synthetic data generation can expand sparse datasets [67, 89]. Regularization techniques (e.g., L1/L2) mitigate the effect of overfitting in model training [90]. Looking forward, the integration of adaptive AI frameworks will redefine precision nutrition by enabling dynamic, real‐time personalization while maintaining model interpretability and clinical relevance, ultimately bridging the gap between sparse data limitations and actionable health insights.

In addition to the main part of multiomics data, including genome and gut microbiome, we highlight that 2D or 3D data(e.g., brain imaging data) will offer valuable new insights into scientific questions in the field of precision nutrition. On the other hand, real‐time monitoring devices (e.g., wearable accelerometers for physical activity and sleep patterns, CGM sensors for continuous interstitial glucose monitoring, as well as mobile phone‐based applications) for real‐time records of dietary intake have been widely used in precision nutrition research [2, 91, 92]. However, many more wearable devices are warranted to generate more comprehensive multimodal data. These devices include, but are not limited to, glass cameras assessing real‐time food and nutrients intakes as well as eating patterns and speed, electronic rings for monitoring heart rate and sleep patterns, user‐friendly wearable EEG devices for physiological monitoring, epidermal patches for continuous monitoring of blood pressure, and in vivo recording electrodes for other biomarkers such as uric acid or alcohol concentrations.

In the era of increasingly sophisticated data collection technologies, comprehensive and longitudinal measurements within individuals have become more feasible, which can enable the applications of n‐of‐1 trials. As discussed above, the n‐of‐1 study design endows scientific rigor to the single‐subject trials and thus holds significant promise for advancing precision nutrition research, particularly as growing numbers of people become interested in self‐monitoring for health purposes. Looking forward, the downstream utilization of multimodal data at the individual level requires more innovative tools or models that are either robust to intracorrelation or capable of explicitly accounting for it.

## 7 Current Challenges and Future Direction

AI has played a pivotal role in processing multimodal data and leveraging it for the development of predictive models, yet significant challenges remain. One major challenge lies in managing the complexity and diversity of data structures in multimodal integration. Integrating numerical data (e.g., omics profiles), time‐series data (e.g., wearable sensor readings), and image data (e.g., MRI scans) requires advanced techniques to handle the heterogeneous formatted data, while noise and high dimensionality often lead to suboptimal model performance. Future progress in AI for science may promote deeper integration of disparate data types to construct digital twins. A second hurdle is that ensuring AI‐based prediction model interpretability poses a significant gap for bench‐to‐bedside translation in the field, especially for precision nutrition management and public guidelines. Currently, decision trees, linear models, LIME, and SHAP not only deliver predictions but also explain why a particular prediction was made [93, 94]. However, higher model performance often means greater model complexity, which often results in reduced interpretability. Therefore, embedding intrinsically interpretable AI frameworks may offer significant potential to uncover novel biomarkers, empowering clinicians to leverage predictive models for personalized dietary guidelines. Lastly, the integration of AI and multimodal data at the individual level for developing individual‐specific predictive models remains underexplored. The n‐of‐1 designs generate relatively limited data per person compared to those at the population level, making it difficult to avoid overfitting while capturing personalized metabolic responses without relying on large population datasets. Future research may focus on developing transfer learning and few‐shot AI frameworks to build up efficient individual model training from n‐of‐1 data. The applications of AI‐powered n‐of‐1 frameworks may thus provide evidence for optimizing dietary interventions tailored to individuals.

## Author Contributions

J.S.Z, Y.H., G.Y., and Y.F. conceived this comprehensive review, and Y.F., K.Z., and Z.M. wrote the manuscript. J.S.Z., Y.H., and G.Y critically revised the manuscript. All authors have approved the final manuscript.

## Conflicts of Interest

The authors declare no conflicts of interest.

## Data Availability

The authors have nothing to report.
